# Usefulness of C-reactive protein as a marker of early post-infarct left ventricular systolic dysfunction

**DOI:** 10.1007/s00011-012-0466-2

**Published:** 2012-03-24

**Authors:** Iwona Swiatkiewicz, Marek Kozinski, Przemyslaw Magielski, Joanna Gierach, Tomasz Fabiszak, Aldona Kubica, Adam Sukiennik, Eliano Pio Navarese, Grazyna Odrowaz-Sypniewska, Jacek Kubica

**Affiliations:** 1 Department of Cardiology and Internal Medicine, Collegium Medicum, Nicolaus Copernicus University, Bydgoszcz, 9 Sklodowskiej-Curie Street, 85-094 Bydgoszcz, Poland; 2 Department of Health Promotion, Collegium Medicum, Nicolaus Copernicus University, Bydgoszcz, Poland; 3 Department of Laboratory Medicine, Collegium Medicum, Nicolaus Copernicus University, Bydgoszcz, Poland

**Keywords:** Acute myocardial infarction, Left ventricular function, Echocardiography, C-reactive protein, Inflammation

## Abstract

**Objective:**

To assess the usefulness of in-hospital measurement of C-reactive protein (CRP) concentration in comparison to well-established risk factors as a marker of post-infarct left ventricular systolic dysfunction (LVSD) at discharge.

**Materials and methods:**

Two hundred and four consecutive patients with ST-segment-elevation myocardial infarction (STEMI) were prospectively enrolled into the study. CRP plasma concentrations were measured before reperfusion, 24 h after admission and at discharge with an ultra-sensitive latex immunoassay.

**Results:**

CRP concentration increased significantly during the first 24 h of hospitalization (2.4 ± 1.9 vs. 15.7 ± 17.0 mg/L; *p* < 0.001) and persisted elevated at discharge (14.7 ± 14.7 mg/L), mainly in 57 patients with LVSD (2.4 ± 1.8 vs. 25.0 ± 23.4 mg/L; *p* < 0.001; CRP at discharge 21.9 ± 18.6 mg/L). The prevalence of LVSD was significantly increased across increasing tertiles of CRP concentration both at 24 h after admission (13.2 vs. 19.1 vs. 51.5 %; *p* < 0.0001) and at discharge (14.7 vs. 23.5 vs. 45.6 %; *p* < 0.0001). Multivariate analysis demonstrated CRP concentration at discharge to be an independent marker of early LVSD (odds ratio of 1.38 for a 10 mg/L increase, 95 % confidence interval 1.01–1.87; *p* < 0.04).

**Conclusion:**

Measurement of CRP plasma concentration at discharge may be useful as a marker of early LVSD in patients after a first STEMI.

## Introduction

Post-infarct left ventricular systolic dysfunction (LVSD) has been identified as a powerful marker of poor prognosis. Its occurrence is associated with an increased risk of cardiac death, re-infarction and re-hospitalization [1–4]. Furthermore, half of patients diagnosed with early post-infarct LVSD subsequently develop chronic heart failure. The prevalence of post-infarct LVSD ranges from 27 to 60 %, depending on the diagnostic criteria applied, therapeutic approach and time when the assessment is made [1–4].

Acute myocardial infarction (MI) provokes a systemic inflammatory response with a release of pro-inflammatory cytokines and enhanced synthesis of C-reactive protein (CRP) [5]. The triggers of cytokine and growth factor release in the setting of MI include mechanical deformation of left ventricle, ischaemia with necrosis, generation of reactive oxygen species, and cytokine self-amplification pathways [6]. Those mediators affect necrosis expansion and scar formation as well as stimulate CRP expression [7]. An increase in CRP plasma concentration in the course of acute MI begins in the first hours following the onset of symptoms, peaks approximately on day 2, and returns to its baseline value after a few weeks [8].

An accumulating body of evidence indicates a close relationship between increased CRP concentrations in patients with MI and excessive mortality in the medium- and long-term follow-up [9–13]. Additionally, evaluation of CRP concentration in this population provides prognostic information independent from the classical risk factors and enhances the value of well-established risk scores [14]. However, the link between CRP and structural and functional cardiac alterations in STEMI patients warrants further investigation.

We therefore set out to assess the usefulness of in-hospital measurement of CRP plasma concentration in comparison to well-established clinical, biochemical and angiographic risk factors as a marker of post-infarct LVSD at discharge in patients with a first ST-segment-elevation myocardial infarction (STEMI) treated with primary percutaneous coronary intervention (pPCI).

## Materials and methods

### Study design and patient characteristics

This study was designed as a single-center prospective observational cohort trial in the setting of first STEMI treated with pPCI. Two hundred and four consecutive patients (156 men and 48 women) meeting the study inclusion and exclusion criteria were enrolled.

The inclusion criteria were as follows: (1) typical stenocardial chest pain of at least 30 min duration, (2) onset of symptoms <12 h before hospital admission and (3) electrocardiographic features of acute STEMI (ST-segment elevation ≥0.1 or ≥0.2 mV in at least two continuous limb or precordial leads, respectively).

The exclusion criteria were: (1) prior coronary revascularization, (2) cardiogenic shock on admission, (3) heart failure (class III or IV according to the New York Heart Association classification), (4) bundle branch block, (5) permanent atrial fibrillation, (6) hemodynamically significant valvular heart disease, (7) primary cardiomyopathy, (8) severe arterial hypertension, (9) creatinine concentration >176.8 mmol/L, (10) the presence of features suggestive of an active inflammatory or neoplastic process on admission, and (11) therapy with steroids, immunosuppressive agents and non-steroidal anti-inflammatory drugs (excluding low doses of aspirin).

The study endpoint was global LVSD, the echocardiographic criterion for which was defined based on previous studies as left ventricular ejection fraction (LVEF) ≤40 % [2, 15, 16]. The patients were divided according to the values of LVEF at discharge into the subgroups with (LVEF ≤40 %) and without (LVEF >40 %) LVSD.

Approval from the local Bioethics Committee at Collegium Medicum in Bydgoszcz was obtained. All patients gave their written, voluntary, informed consent for participation in the study.

### Pharmacotherapy

At the first contact with health care providers immediately after the diagnosis of STEMI, all patients were pre-treated with an intravenous bolus of unfractionated heparin (70 IU/kg, up to 5,000 IU) and oral loading doses of clopidogrel (600 mg) and aspirin (300 mg). At the catheterization laboratory a second dose of unfractionated heparin was administered intra-arterially in a weight-adjusted manner (up to 100 IU/kg) or under activated clotting time guidance (to the target range of 200–250 s) when abciximab was intended. Abciximab was given at the discretion of the invasive cardiologist. Throughout the study period clopidogrel and aspirin 75 mg q.d. were continued in all patients. Concomitant medications in the majority of patients included perindopril and long-acting metoprolol in doses adjusted for resting heart rate and blood pressure, and simvastatin 40 mg q.d. (Table 1). Additionally, 17 (8.3 %) patients were treated with spironolactone while 13 (6.4 %) participants received non-potassium-sparing diuretics.

**Table 1:** Demographic and clinical characteristics of the study population

Variable	Overall study population (*n* = 204)	Patients with LVSD (*n* = 57)	Patients without LVSD (*n* = 147)	*p* for comparison between groups with and without LVSD
Age (years)	57.0 ± 9.2	59.0 ± 8.7	56.2 ± 9.3	<0.05
Gender (male/female), *n* (%)	156/48 (76.5/23.5)	43/14 (75.4/24.6)	113/34 (76.9/23.1)	NS
Anterior wall STEMI, *n* (%)	89 (43.6)	52 (91.2)	37 (25.2)	<0.001
Time from onset of pain to balloon (min)	238.2 ± 151.1	233.8 ± 150.0	244.9 ± 150.8	NS
Risk factors for coronary artery disease				
Body mass index (kg/m^2^)	26.8 ± 3.9	27.9 ± 4.3	26.4 ± 3.6	<0.01
Hypertension, *n* (%)	84 (41.2)	31 (54.4)	53 (36.1)	<0.02
Diabetes mellitus, *n* (%)	37 (18.1)	15 (26.3)	22 (15.0)	NS
Current or ex-smoker, *n* (%)	134 (65.7)	34 (59.7)	100 (68.0)	NS
LDL cholesterol (mmol/L)	3.87 ± 1.02	3.92 ± 1.09	3.85 ± 1.0	NS
HDL cholesterol (mmol/L)	1.37 ± 0.29	1.27 ± 0.26	1.38 ± 0.29	<0.05
Triglycerides (mmol/L)	1.33 ± 1.08	1.41 ± 0.86	1.30 ± 1.15	<0.05
Cardiological history				
Angina proceeding to myocardial infarction, *n* (%)	86 (42.2 %)	26 (45.6 %)	60 (40.8 %)	NS
Heart failure prior to MI (I or II class according to the NYHA classification), *n* (%)	7 (3.5)	3 (5.3)	4 (2.7)	NS
Medical treatment				
Long-acting metoprolol	202 (99.0 %)	56 (98.2 %)	146 (99.3 %)	NS
Perindopril	200 (98.0 %)	55 (96.5 %)	145 (98.6 %)	NS
Simvastatin	203 (99.5 %)	57 (100.0 %)	146 (99.3 %)	NS
Spironolactone	17 (8.3 %)	10 (17.5 %)	7 (4.8 %)	<0.004
Non-potassium-sparing diuretics	13 (6.4 %)	8 (14.0 %)	5 (3.4 %)	<0.006

*LVSD* left ventricular systolic dysfunction, *MI* myocardial infarction, *NYHA* New York Heart Association, *STEMI* ST-segment-elevation myocardial infarction

### Coronary angiography and pPCI

Coronarography and pPCI were performed using a standard femoral approach. The use of aspiration thrombectomy during the intervention was left to the operator’s discretion. Intracoronary stents were routinely implanted. Coronary artery stenosis was measured with quantitative coronary angiography. Epicardial coronary flow was assessed according to the Thrombolysis in Myocardial Infarction (TIMI) score and TIMI frame count (TFC), and myocardial perfusion according to the TIMI Myocardial Perfusion Grade (TMPG).

### Echocardiography

Transthoracic echocardiographic recordings employing the Doppler technique were acquired before discharge using a Philips SONOS 7500 Ultrasound System, according to the protocol recommended by the American Society of Echocardiography [17]. Echocardiographic recordings were assessed offline by two independent experienced echocardiographers blinded to the values of biomarker measurement. Measurements are reported as the average of three consecutive cardiac cycles. The echocardiographic results obtained by echocardiographers were averaged. The inter- and intra-observer coefficients of variation for LVEF assessed in the first 50 patients were below 5.0 and 2.5 %, respectively.

We assessed the sizes of the heart chambers, myocardium wall thickness and the following parameters of left ventricular systolic function: (1) LVEF measured with the biplane method of discs in four- and two-chamber views, and (2) wall motion score index (WMSI), derived as a sum of all scores divided by the number of segments visualized, implementing the 16-segment model of left ventricle segmentation and assigning a score of 1, 2, 3, or 4 points for normokinesis, hypokinesis, akinesis and dyskinesis, respectively [18]. Left ventricular mass was calculated according to the Devereux formula [19]. Measurements of peak systolic mitral annular velocities were obtained for four basal segments of the left ventricle (septal, lateral, inferior and anterior) using pulsed tissue Doppler echocardiography with the Doppler gate targeted at the junction between the left ventricle walls and the mitral annulus in four- and two-chamber views. The average peak systolic mitral annular velocity (*S*′) and an average septal and lateral peak systolic mitral annular velocity (*S*″) were obtained.

### Blood sampling and laboratory analyses

Peripheral venous blood samples were collected using ethylenediaminetetraacetic acid tubes. After being centrifuged, the plasma was stored at −80 °C until analyzed.

CRP plasma concentrations were measured with an ultra-sensitive latex immunoassay (CRP Vario test, analyzer: ARCHITECT ci8200, Abbott) at admission, 24 h after admission and at discharge. B-type natriuretic peptide (BNP) plasma concentration was measured with a chemiluminescent microparticle immunoassay (analyzer: ARCHITECT ci8200) at admission and at discharge. The limits of detection for CRP and BNP were 0.1 mg/L and 10 pg/L, respectively. The intra-assay coefficients of variation were below 2.0 % for CRP and below 5.0 % for BNP, while the inter-assay coefficients of variation were below 1.0 % for CRP and below 5.0 % for BNP, respectively.

### Statistical analysis

Due to major advances in STEMI management resulting in improved survival and lower prevalence of post-infarct LVSD along with reductions in mean CRP values in STEMI patients in recent years, we decided to perform an internal pilot study of the first 50 patients for estimating the final sample size. To compensate for the potential loss of patients due to withdrawal of consent or other reasons, we enrolled an additional patient. LVSD was present in 15 (29.4 %) subjects. CRP concentrations in the first 51 patients assessed for the overall population and for patients with and without LVSD were, respectively (1) on admission 2.6 ± 2.1, 2.7 ± 1.9 and 2.6 ± 2.1 mg/L, (2) at 24 h after admission 15.8 ± 14.1, 25.6 ± 19.0 and 11.7 ± 9.7 mg/L, and (3) at discharge 16.5 ± 16.2, 24.0 ± 19.9 and 13.4 ± 13.5 mg/L. Based on these results and assuming a two-sided alpha value of 0.05, we calculated that enrolment of 200 patients would provide a 99.9 and 98.9 % power to demonstrate significant differences in CRP concentrations between patients with and without LVSD at 24 h after admission and at discharge, respectively. We decided to obtain such high power to be able to perform credible multivariate analyses.

Continuous variables were presented as mean values ± standard deviations. The Shapiro–Wilk test was used to demonstrate whether the investigated variables were normally distributed. Depending on the presence or absence of normal distribution, inter-group comparisons were performed with Student’s *t* test for independent samples or the Mann–Whitney unpaired rank sum test, whereas Student’s *t* test for paired samples or the Wilcoxon matched-paired rank sum test were applied for comparisons within the groups. Categorical variables were compared using the *χ* ^2^ test with Yates’ correction if needed.

Univariate and multivariate logistic regression models were used to identify markers of LVSD. Relations between the investigated variables and the likelihood of LVSD were estimated with the use of odds ratios (ORs) and their 95 % confidence intervals (95 % CIs). The optimal cut-off points were determined using receiver operator characteristic (ROC) curve analysis.

The impact of numerous variables on a quantitative variable was assessed using the multiple regression model.

A two-sided difference was considered significant at *p* < 0.05. The statistical analysis and sample size calculation were carried out using the Statistica 10.0 package (StatSoft, Tulsa, OK, USA).

## Results

### Clinical, echocardiographic and angiographic assessment

LVSD at discharge was present in 57 (27.9 %) patients in our study. Patients with LVSD when compared to those with LVEF >40 % were older, had much more frequent anterior location of STEMI, and were more likely to be overweight, dyslipidemic and hypertensive (Table 1).

The subgroup with LVEF ≤40 % had significantly higher diameters of left atrium and left ventricle, bigger systolic and diastolic left ventricular volumes and greater left ventricle mass than patients with LVEF >40 % (Table 2). Similarly, in the former group we observed significantly higher values of WMSI and markedly lower average peak systolic mitral annular velocity and average septal and lateral peak systolic mitral annulus velocity indicating more impaired regional and longitudinal left ventricular systolic function.

**Table 2:** Angiographic, echocardiographic and biochemical characteristics of study population

Variable	Overall study population (*n* = 204)	Patients with LVSD (*n* = 57)	Patients without LVSD (*n* = 147)	*p* for comparison between groups with and without LVSD
Angiographic indices				
IRA: LAD/non-LAD, *n* (%)	93 (45.6)/111 (54.4)	52 (91.2)/5 (8.8)	41 (27.9)/106 (72.1)	<0.001
Multivessel coronary artery disease, *n* (%)	123 (60.3)	38 (66.7)	85 (57.8)	NS
Stenosis in IRA in QCA (%)				
Before pPCI	93.9 ± 9.5	95.5 ± 9.2	93.3 ± 9.5	0.035
After pPCI	11.7 ± 10.1	10.6 ± 8.2	12.1 ± 10.7	NS
TFC in IRA (frames/s)				
Before pPCI	74.6 ± 33.5	83.1 ± 29.2	71.3 ± 34.6	0.021
After pPCI	25.8 ± 17.9	28.0 ± 15.2	24.9 ± 18.9	0.017
TIMI 3 flow in IRA, *n* (%)				
Before pPCI	58 (28.4)	6 (10.5)	52 (35.4)	<0.001
After pPCI	190 (93.1)	50 (87.7)	140 (95.3)	NS
TMPG 3 after pPCI, *n* (%)	94 (46.1)	28 (49.1)	66 (44.9)	NS
Patients with implanted stents, *n* (%)	202 (99.0)	57 (100)	145 (98.6)	NS
Patients with implanted DES, *n* (%)	4 (2.0)	2 (3.6)	2 (1.4)	NS
Abciximab use, *n* (%)	50 (24.5)	23 (41.1)	27 (18.6)	<0.001
Echocardiographic indices				
LA (mm)	39.7 ± 4.5	41.1 ± 5.3	39.1 ± 4.1	0.029
LVEDd (mm)	48.7 ± 5.5	51.4 ± 5.1	47.6 ± 5.3	<0.001
LVESd (mm)	33.8 ± 4.8	36.8 ± 5.1	32.6 ± 4.2	<0.001
LVMI (g/m²)	115.7 ± 25.2	136.1 ± 24.7	107.8 ± 20.5	<0.001
LVEDVI (mL/m^2^)	53.0 ± 12.6	60.5 ± 14.5	50.1 ± 10.5	<0.001
LVESVI (mL/m^2^)	29.7 ± 9.5	38.5 ± 10.5	26.3 ± 9.5	<0.001
WMSI (points)	1.6 ± 0.2	1.8 ± 0.1	1.5 ± 0.2	<0.001
*S*′ (cm/s)	7.2 ± 1.4	6.1 ± 1.1	7.6 ± 1.4	<0.001
*S*″ (cm/s)	7.2 ± 1.5	6.1 ± 1.1	7.6 ± 1.4	<0.001
Biochemical parameters				
Creatinine (μmol/L)	85.0 ± 15.7	87.6 ± 16.7	84.0 ± 15.2	NS
Admission glucose (mmol/L)	8.46 ± 3.05	9.77 ± 4.24	7.96 ± 2.26	0.002
HbA_1c_ (%)	6.3 ± 1.1	6.6 ± 1.4	6.2 ± 1.0	NS
TnI_max_ (ng/mL)	32.1 ± 19.6	43.4 ± 14.3	27.7 ± 19.7	<0.001
CK-MB_max_ (U/L)	120.5 ± 81.6	158.1 ± 86.3	105.5 ± 74.8	<0.001
Leukocyte count at admission (10^3^ per μL)	11.2 ± 3.0	11.6 ± 2.8	11.1 ± 3.0	NS
Leukocyte count 24 h after admission (10^3^ per μL)	10.3 ± 2.6	11.5 ± 3.0	9.9 ± 2.2	<0.001
BNP at admission (pg/mL)	87.1 ± 140.0	136.8 ± 230.4	67.9 ± 74.6	0.002
BNP at discharge (pg/mL)	205.0 ± 260.2	401.0 ± 386.0	129.0 ± 127.0	<0.001

Echocardiographic indices are derived from 2D and Doppler echocardiography and tissue Doppler echocardiography at hospital discharge

*BNP* B-type natriuretic peptide, *CK-MB*_*max*_ maximal activity of isoenzyme MB of creatine kinase, *DES* drug-eluting stent, *IRA* infarct-related artery, *LA* left atrium; *LAD* left anterior descending artery, *LVEDd* left ventricular end-diastolic diameter, *LVEDVI* left ventricular end-diastolic volume index, *LVESd* left ventricular end-systolic diameter, *LVESVI* left ventricular end-systolic volume index; *LVMI* left ventricle mass index, *LVSD* left ventricular systolic dysfunction, *pPCI* primary percutaneous coronary intervention, *TnI*_*max*_ maximal concentration of troponin I, *TIMI* Thrombolysis in Myocardial Infarction score, *TFC* TIMI frame count, *TMPG* TIMI Myocardial Perfusion Grade, *S′* average peak systolic mitral annular velocity, *S″* average septal and lateral peak systolic mitral annulus velocity, *WMSI* wall motion score index

In accordance with the dominant anterior location of STEMI in patients with LVSD, the culprit lesion was found much more frequently in the left descending artery in this subgroup than in patients without LVSD (Table 2). Furthermore, patients with LVEF ≤40 % at discharge presented with considerably less favourable pre-pPCI angiographic indices and interventional cardiologists were more likely to administer abciximab in this population than in patients with LVEF >40 % at discharge (Table 2). Although in the majority of patients pPCI resulted in a complete restoration of epicardial blood flow in the infarct-related artery, the incidence of complete reperfusion in the area of STEMI denoted by TMPG 3 was below 50 % in both subgroups.

### Biomarkers

Patients with LVSD at discharge when compared to those with LVEF >40 % presented with a significantly higher maximal concentration of troponin I, markedly elevated plasma glucose on admission and noticeably increased white blood cell count at 24 h after admission (Table 2).

**Fig. 1:**
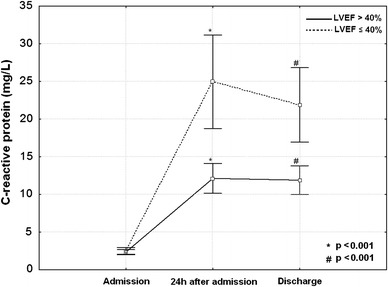
C-reactive protein plasma concentrations as mean values and standard deviations on admission, 24 h after admission and at hospital discharge in patients with and without early post-infarct left ventricular systolic dysfunction. *LVEF* left ventricular ejection fraction

**Fig. 2:**
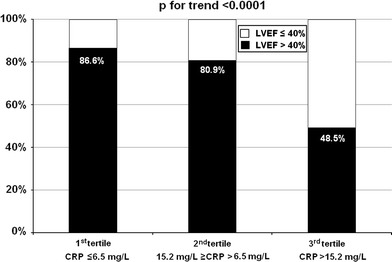
Incidence of global left ventricular systolic dysfunction at hospital discharge according to tertiles of C-reactive protein plasma concentration 24 h after admission. *CRP* C-reactive protein, *LVEF* left ventricular ejection fraction

**Fig. 3:**
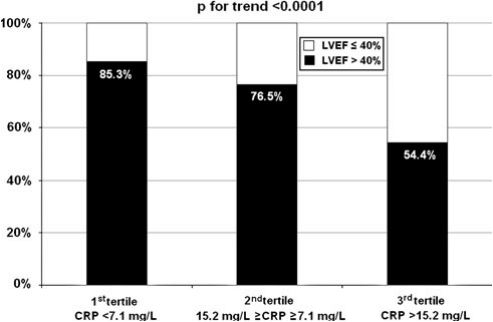
Incidence of global left ventricular systolic dysfunction at hospital discharge according to tertiles of C-reactive protein plasma concentration at discharge. *CRP* C-reactive protein, *LVEF* left ventricular ejection fraction

CRP plasma concentration rose steeply during the first 24 h of hospitalization (*p* < 0.001) and persisted elevated at discharge, mainly in patients with LVSD (Fig. 1). As shown in Figs. 2 and 3, the prevalence of LVSD was significantly increased across increasing tertiles of CRP concentration, both at 24 h after admission (cut-off values of ≤6.5 and >15.2 mg/L for the lower and upper tertile, respectively) and at discharge (cut-off values of <7.1 and >15.2 mg/L for the lower and upper tertile, respectively).

BNP concentration increased during hospitalization in all patients (*p* < 0.001). However, its markedly higher values were observed in the group with LVEF ≤40 % at both time points, allowing early identification of patients prone to developing global LVSD (Table 2).

### Markers of LVSD in multivariate analysis

Markers of LVSD at discharge revealed by the univariate logistic regression analysis are presented in Table 3. The final model of multivariate logistic regression analysis found the anterior location of STEMI, maximal concentration of troponin I and CRP plasma concentration at discharge to be independent factors associated with early LVSD. Surprisingly, despite a very good ability to distinguish between patients with and without LVSD and an excellent discriminating value in the univariate analysis, BNP concentration failed to be an independent marker of LVSD in the multivariate analysis. Similarly, when adjusted for CRP concentration the leukocyte count was no longer associated with LVSD in the multivariate analysis.

**Table 3:** Markers of early left ventricular systolic dysfunction in univariate and multivariate analyses

	OR	95 % CI	*p*
Univariate analysis			
Anterior vs. non-anterior wall STEMI	30.92	11.41–83.75	<0.0001
Heart failure prior to MI (I or II class according to the NYHA classification)	7.52	3.00–18.83	<0.0002
Body mass index (for a 10 kg/m^2^ increase)	2.76	1.22–6.23	<0.02
Hypertension	2.11	1.13–3.95	<0.02
Diabetes mellitus	2.03	0.96–4.29	0.066
BNP at discharge (for a 100 pg/mL increase)	1.89	1.48–2.43	<0.0001
TnI_max_ (for a 10 ng/mL increase)	1.67	1.35–2.06	<0.0001
BNP at admission (for a 100 pg/mL increase)	1.61	1.14–2.28	<0.01
CRP 24 h after admission (for a 10 mg/L increase)	1.60	1.27–2.00	<0.0001
CRP at discharge (for a 10 mg/L increase)	1.55	1.24–1.93	<0.0002
Age (for a 10-year increase)	1.38	0.99–1.94	0.056
CRP at admission (for a 10 mg/L increase)	1.37	0.27–7.03	0.70
Leukocyte count 24 h after admission (for a 10^3^ per μL increase)	1.30	1.14–1.48	<0.002
HbA1c (for a 1 % increase)	1.29	1.00–1.66	<0.05
Admission glycaemia (for a 1 mmol/L increase)	1.21	1.08–1.35	<0.001
CK-MB_max_ (for a 10 U/L increase)	1.08	1.04–1.13	<0.0002
Leukocyte count at admission (for a 10^3^ per μL increase)	1.06	0.96–1.18	0.25
Multivariate analysis			
Anterior vs. non-anterior wall STEMI	26.67	9.42–75.52	<0.001
TnI_max_ (for a 10 ng/mL increase)	1.39	1.10–1.77	<0.007
CRP at discharge (for a 10 mg/L increase)	1.38	1.01–1.87	<0.04

Univariate analysis shows demographic, clinical, angiographic and biochemical parameters from Tables 1 and 2 with a *p* value ≤0.1 as well as CRP and leukocyte count independently of a *p* value

*BNP* B-type natriuretic peptide, *CI* confidence interval, *CK-MB*_*max*_ maximal activity of isoenzyme MB of creatine kinase, *CRP* C-reactive protein, *MI* myocardial infarction, *NYHA* New York Heart Association, *OR* odds ratio, *STEMI* ST-segment-elevation myocardial infarction, *TnI*_*max*_ maximal concentration of troponin I

### Optimal cut-off values for the detection of LVSD

The ROC curve analysis assessing the diagnostic accuracy for the detection of LVSD at discharge revealed optimal cut-off values of 17.5 mg/L for CRP at discharge (sensitivity 49.1 %, specificity 83.7 %, positive value for LVSD detection 53.8 %, negative value for LVSD detection 80.9 %) and 46.3 ng/mL for maximal troponin I concentration (sensitivity 80.7 %, specificity 63.9 %, positive value for LVSD detection 46.5 %, negative value for LVSD detection 89.5 %). Areas under the ROC curves for CRP at discharge and the maximal troponin I concentration were 0.695 (95 % CI 0.627–0.757) and 0.779 (95 % CI 0.716–0.834), respectively. Comparison of the ROC curves for both biomarkers in terms of their diagnostic accuracy demonstrated the superiority of the maximal troponin I concentration over CRP value at discharge of a borderline significance (*p* = 0.06).

### Determinants of CRP concentration

We applied the multiple regression model to determine which of the demographic, clinical, angiographic and biochemical parameters listed in Tables 1 and 2 affect CRP concentration at discharge. Increased BNP values at discharge, high maximal concentration of troponin I and elderly age were independently associated with elevated CRP concentration at discharge (Table 4).

**Table 4:** Impact of demographic, clinical, angiographic and biochemical variables from Tables 1 and 2 on CRP concentration at discharge in the multiple regression model

	Beta coefficient	Beta coefficient standard error	Direction component beta	Direction component beta standard error	*p*
Model characteristics: *R* = 0.50; *R* ^2^ = 0.25; *p* < 0.00001					
Intercept			−0.80	0.61	
Age (for a 10-year increase)	0.15	0.06	0.24	0.10	<0.02
TnI_max_ (for a 10 ng/mL increase)	0.21	0.07	0.15	0.05	<0.002
BNP at discharge (for a 100 pg/mL increase)	0.35	0.07	0.20	0.04	<0.00001

*CRP* C-reactive protein, *TnI*_*max*_ maximal concentration of troponin I

## Discussion

The main finding of our study is a clear relationship between in-hospital CRP plasma concentrations and the development of early post-infarct LVSD in patients undergoing pPCI for a first STEMI. In the homogeneous population treated in line with contemporary standards, CRP maintained its discriminating value for early post-infarct LVSD detection, even when adjusted for well-established clinical, biochemical and angiographic risk factors. Of interest, CRP concentration at discharge identified patients with early post-infarct LVSD better than leukocyte count and BNP concentration. When CRP value at discharge was incorporated into the multivariable model, both leukocyte count and BNP concentration lost their discriminating values.

The rapid rise in CRP concentration within 24 h of symptom onset persisting until discharge reflects the severity of the inflammatory reaction within the infarcted area. We found maximal concentration of troponin I, BNP value at discharge and patient’s age to be independent determinants of the magnitude of the inflammatory response assessed by CRP concentration at discharge.

We selected CRP as a sensitive, well-standardized biomarker with proven value in terms of clinical risk stratification in cardiovascular medicine. Increased CRP concentrations were linked with an excess risk of death, heart failure, cardiac rapture, ventricular aneurysmal formation, and thrombus formation in MI survivors [9–13, 20, 21]. Incorporation of CRP into the Global Registry of Acute Coronary Events risk score further improved its predictive power [14]. Therefore, combining these data with our results, we and other authors believe that CRP might be a simple and reliable marker for the magnitude of the inflammatory response to myocardial necrosis, providing prognostic information in STEMI patients [10, 22].

To the best of our knowledge, this is the first study linking CRP concentration and post-infarct LVSD conducted exclusively in a STEMI population, in a subset of patients with severely deteriorated epicardial blood flow and a large area of infarcted myocardium. Moreover, we applied strict inclusion criteria and numerous exclusion criteria to eliminate many of the potential confounders in our study. Almost three-quarters of our patients had an impaired TIMI flow on the initial angiogram while the median maximal concentration of troponin I in the study participants was 100-fold higher than the detection limit for MI in our laboratory. Previous studies in this field mostly recruited patients with a broad spectrum of acute coronary syndromes [23–25]. This fact seems to be of paramount importance when considering different mechanisms responsible for CRP synthesis as well as dissimilar magnitudes of CRP release in MI versus unstable angina [22, 26]. In stable and unstable coronary artery disease, elevated CRP reflects inflammation in the vascular bed or vulnerability of unstable plaques in contrast to MI, where the inflammatory response to myocardial necrosis dominates.

As far as we know a study conducted by Aggelopoulos et al. [23] is the only one to date investigating the relationship between CRP concentration and the presence of LVSD in patients treated for acute coronary syndromes. Other studies [24, 25, 27] in this field assessed LVEF as a continuous variable. Aggelopoulos et al. [23] showed that an increase in CRP plasma concentration during 12 h after admission as high as 10 mg/L was an independent predictor of a 6 % augmentation of risk for LVSD at discharge. However, the results may be at least partially flawed by important limitations such as retrospective case–control design, history of coronary artery disease in almost half of the study participants, inclusion of patients with unstable angina, exclusion of patients with LVEF between 40 and 50 % from the analysis and application of low-sensitivity CRP assay.

Studies linking CRP concentrations and LVEF in MI survivors gave conflicting results [8, 25, 28]. Ørn et al. demonstrated that early measurement of CRP (viz. at 2 days and 1 week) significantly predicted LVEF assessed by cardiac magnetic resonance after 2 months in a small but well-designed study of STEMI patients [28]. Similarly, Uehara et al. [8] found a significant inverse correlation between the peak of in-hospital CRP concentration and LVEF at 1 month after STEMI. It is likely that various confounders present in many of the studies addressing this issue might lead to unreliable conclusions. For example, heterogeneity of the investigated population, with a substantial proportion of unstable angina patients, might have been the reason for the lack of correlation between CRP concentration and LVEF in the study by Brunetti et al. [25]. Furthermore, the timing of CRP assessment in the course of MI seems to be crucial for its diagnostic value. In our study, while absent on admission (with 4 h mean delay between symptom onset and admission), significantly higher CRP concentrations were present 24 h after admission and at discharge in patients with early LVSD. Corresponding to our results, Arruda-Olson et al. [24] observed comparable values of LVEF and WMSI in tertiles of CRP evaluated at a median of 6.1 h after symptom onset. On the other hand, Suleiman et al., who measured CRP 12–24 h after symptom onset, found inverse relations between CRP concentration and both LVEF and WMSI values in patients with acute MI [10].

Finally, it remains an unsolved issue whether CRP directly contributes to post-infarct LVSD and may be a potential therapeutic target, or if it just reflects an increased risk for unfavourable outcome as a bystander marker [29, 30]. A large body of basic scientific evidence suggests that CRP possesses both pronecrotic and proatherogenic features. Firstly, CRP binds to phosphocholine groups of necrotic myocardial cell membranes, leading to complement activation and thus promoting further inflammatory response, injury of myocardial cells and expansion of necrosis [31, 32]. Secondly, elevated endogenous CRP was associated with an increase in ischemia/reperfusion injury in a rabbit model [33]. Thirdly, increased C-reactive protein expression exacerbated LVSD and remodeling after MI in a mouse model [34]. This deleterious effect of CRP on post-MI left ventricular remodeling was related to increased apoptotic rates, macrophage infiltration, monocyte chemotactic protein-1 expression and matrix metalloproteinase-9 activity in the border zone. Additionally, CRP reduces bioavailability of nitric oxide, which in turn suppresses angiogenesis [35]. CRP also inhibits endothelial progenitor cell differentiation, function and survival [36].

### Limitations of the study

Major limitations of our study include short-term follow-up and lack of concomitant assessment of cytokines and growth factors. Furthermore, due to early achievement of reperfusion, our patients had relatively well-preserved left ventricular systolic function. Additionally, we did not account in our calculations for diurnal and seasonal variations in CRP concentration. Despite encouraging results obtained in our study, further efforts are warranted to confirm their clinical significance and to fully explain the mechanisms through which augmentation of the inflammatory process contributes to the occurrence of LVSD and subsequently to the development of heart failure following a STEMI.

## Conclusions

The measurement of CRP plasma concentration at discharge may be useful as a marker of early LVSD in patients after a first STEMI.
